# Investigating Reading Enjoyment in Adults With Dyslexia

**DOI:** 10.1002/dys.1803

**Published:** 2025-02-26

**Authors:** Hannah Jones, Amrita Bains, Laura Randall, Carina Spaulding, Jessie Ricketts, Saloni Krishnan

**Affiliations:** ^1^ Department of Psychology, Royal Holloway University of London Surrey UK; ^2^ Division of Psychology and Language Sciences, UCL London UK; ^3^ Department of Experimental Psychology University of Oxford UK; ^4^ The Reading Agency London UK

**Keywords:** dyslexia, literacy, reading, reading comprehension

## Abstract

Previous studies have suggested that adults with dyslexia do not enjoy reading, based on data from trait‐based questionnaires. This study uses state‐based measures of reading to offer greater insight into different aspects of motivation, including liking and wanting. In a new paradigm sensitive to dynamic changes in reading enjoyment, adults with dyslexia (*n* = 59) and without dyslexia (*n* = 59) read 24 book extracts, rated their enjoyment and answered a question about the extract. Subsequently, participants decided whether to accept a cost (e.g., 3–6 s wait) to read the next paragraph. We also collected traditional trait‐based measures of enjoyment. While neurotypical adults had higher trait‐based reading enjoyment, average state‐based reading enjoyment did not differ between groups. The relationship between high enjoyment states and subsequent benefits was altered in dyslexia. While heightened states of enjoyment increased the likelihood of continuing to read, this was attenuated in adults with dyslexia. In neurotypical adults, high states of enjoyment increased the likelihood of answering the question about the text correctly; this relationship did not hold in adults with dyslexia. Our findings shed light on how links between intrinsic value and subsequent motivation are altered in adults with dyslexia, suggesting that reading enjoyment can drive reading engagement but not comprehension.

## 1 Investigating Reading Enjoyment in Adults With Dyslexia

Dyslexia is a neurodevelopmental condition characterised by difficulties with decoding text and spelling (Snowling, Hulme, and Nation 2020), with a prevalence of 3%–7% (Zaccoletti, Altoè, and Mason 2020). Those with dyslexia often report more early unrewarding reading experiences (Humphrey 2002; Humphrey and Mullins 2002; van Bergen, Vasalampi, and Torppa 2021) and low reading enjoyment (Johnson 2018; Polychroni, Koukoura, and Anagnostou 2006; Soriano‐Ferrer and Morte‐Soriano 2017). A lack of engagement can establish a vicious cycle, as reading more is linked with better learning (Anderson, Wilson, and Fielding 1988; Anmarkrud and Bråten 2009; van Bergen, Vasalampi, and Torppa 2021) and well‐being (Billington 2015; Boyes et al. 2016; Clark and Teravainen‐Goff 2018; Humphrey 2002; Humphrey and Mullins 2002; Wilmot et al. 2023; Zuppardo et al. 2023). Here, for the first time, we use an experimental design to investigate *states* of reading enjoyment in adults with dyslexia. This is a crucial step to understand why motivation differs in individuals with dyslexia. We ask whether adults with dyslexia experience similar states of reading enjoyment as neurotypical adults, and whether such rewarding reading experiences lead to the same benefits as seen in neurotypical adults.

State‐based measures have the potential to provide critical insight into the processes that underpin motivation in adults with dyslexia. State‐based (or situational) factors, such as the texts people are provided, are potentially modifiable and could be used to create better interventions or reading contexts. Yet, to date, most studies that measure motivation have used a trait‐based approach, usually relying on a single self‐report question, for example, ‘Do you like reading?’ (van Bergen et al. 2023). These studies show that children and adults with dyslexia show lower reading enjoyment and choose to read less (Johnson 2018; Polychroni, Koukoura, and Anagnostou 2006; Soriano‐Ferrer and Morte‐Soriano 2017; van Bergen, Vasalampi, and Torppa 2021). However, trait‐based measures quantify a person's general attitude towards reading (Zaccoletti, Altoè, and Mason 2020), closely reflecting self‐perception of reading achievements (Davis et al. 2018; Eccles, Wigfield, and Schiefele 1998). Adults with dyslexia have experienced a long history of reading difficulties, which is a potential confound as they are likely to have lower self‐efficacy. Some studies have used teacher ratings (Humphrey 2002; Humphrey and Mullins 2002; Soriano‐Ferrer and Morte‐Soriano 2017), but a diagnosis of dyslexia could influence teacher's expectations of the child. In practice, having dyslexia and being an avid reader are not mutually exclusive (Dobson Waters and Torgerson 2021; Fink 1995; Madriaga 2007; Moojen et al. 2020), indicating that having dyslexia is not a permanent barrier to enjoying reading. Capturing the dynamic changes in enjoyment that influence an individual's decision to start or to continue to read (Davis et al. 2018; Eccles, Wigfield, and Schiefele 1998) could offer important insight into motivation.

In a recent study, we presented neurotypical adults with 40 book synopses, asking them to rate their enjoyment of these different texts (Bains et al. 2023a). This allowed us to measure states of enjoyment over a diverse set of texts, capturing situation‐specific enjoyment. We also tapped a distinction made in the decision science literature between measures of enjoyment or ‘liking’ and a motivation‐specific behaviour or ‘wanting’ (Berridge and Robinson 2003). While these are correlated in most situations, they can be disassociated—for instance, we may like something but not be willing to pursue it. In the decision sciences, such pursuit is considered a cost. The cost of a reward reduces motivation, so a reward is particularly desirable if a person is willing to accept a temporal, monetary or effort‐based cost to obtain it (Blain and Sharot 2021; Kang et al. 2009; Marvin and Shohamy 2016). In Bains et al. (2023a), we assessed whether enjoyment of a synopsis predicted whether adult readers waited for 3–6 s to see an associated book cover (Bains et al. 2023a). High enjoyment was associated with a propensity to accept this temporal cost, as well as better comprehension of the extract (Bains et al. 2023a). This demonstrated that we could establish the value of specific texts and link this to future reading‐relevant behaviour. However, in this study, we did not test individuals with dyslexia, who are likely to differ in the intrinsic reward value they assign to text as well as experience heightened costs. Given their challenges with reading, individuals with dyslexia might experience overall lower enjoyment of texts and demonstrate lower motivation to read further. However, they may also show greater motivation and comprehension when they experience enjoyment. In this case, our findings would strongly indicate a need for reading programmes to incorporate enjoyment into their design.

We tested adults with and without dyslexia using a modified version of the willingness‐to‐wait design employed by Bains and colleagues (2023a). In our version, participants incurred a temporal cost if they wanted to read more (in the previous study, participants waited to view a book cover). This modification was made to better simulate real‐world decisions about continuing to read, as participants who prefer visual stimuli may be more likely to wait for book covers (Oxford 1992). We expected to replicate our previous findings that heightened states of enjoyment would be associated with choosing to read more and improved comprehension (in both neurotypical adults and in adults with dyslexia).

## 2 Methods

### 2.1 Pre‐Registration

We pre‐registered our hypotheses on the Open Science Framework (https://doi.org/10.17605/OSF.IO/T95H6).

### 2.2 Power Analysis

To determine sample sizes, a power analysis was conducted using the SimR package (Green and MacLeod 2016). Simulations were based on a previous study (Bains et al. 2023a), with 37 participants and 24 items. Using an alpha of 0.05, 80% power, we required 20 participants to detect a similar effect of enjoyment on waiting as observed in Bains et al. (2023a). To have 80% power to detect an effect of enjoyment on comprehension, we required 60 participants. Our target sample was therefore 120 (60 with dyslexia and 60 neurotypical adults).

### 2.3 Participants

We recruited 123 participants (60 neurotypical controls and 63 adults with dyslexia). Participants were recruited through university mailing lists, dyslexia organisations and social media. A small proportion were recruited via Prolific (https://www.prolific.com/). Participants were between the ages of 18 and 30 years, had normal or corrected‐to‐normal vision and hearing and were native English speakers. Participants with additional neurodevelopmental conditions (epilepsy, ADHD, autism and genetic disorders) or any other learning disorders were excluded. One neurotypical (male) participant and 4 (3 females; 1 male) participants with dyslexia were excluded on this basis, leaving us with a final sample of 118.

### 2.4 Materials

#### 2.4.1 Abbreviated Adult Reading History Questionnaire (ARHQ‐Brief)

The Abbreviated Adult Reading History Questionnaire‐Brief (AHRQ‐Brief) (Feng et al. 2022) comprised six items assessing reading history. Participants rated each question on a 4‐point Likert scale. A previous study has shown that scores on the AHRQ‐Brief are strongly correlated with a composite reading ability score (Feng et al. 2022).

#### 2.4.2 Sentence Verification Task

The Sentence Verification task (Garvin and Krishnan 2022) assessed reading fluency and comprehension. Participants were asked to read and verify the truthfulness of 80 sentences based on their real‐world knowledge (e.g., ‘Cars drive on water’). Each sentence was on screen for a maximum of 3 s. Participants were given 90 s to score the maximum number of points. Sentence length increased across the five blocks and became more difficult to read (Garvin and Krishnan 2022). Participants received 1 point for each sentence they correctly verified, with a maximum possible score of 80. The task can be viewed on Gorilla Open Materials (https://app.gorilla.sc/openmaterials/237000).

#### 2.4.3 Willingness‐to‐Wait Task

The design of the willingness‐to‐wait task was akin to Bains et al. (2023a). Participants were presented with an extract from a book. To reduce the pressure on readers with dyslexia, reading was self‐paced. Following this, participants were asked to rate their enjoyment of the extract on a scale of 1–9. Participants were also asked if they were familiar with the book. Participants then answered a question about the book. Finally, participants decided whether to wait between 3 and 6 s to read more of the same book. There was no time penalty imposed if participants wanted to skip to the next trial. Participants were made aware that the choice to wait was entirely voluntary, and there was no reward for waiting.

Our stimuli were from the Quick Reads produced by The Reading Agency (https://readingagency.org.uk/get‐reading/our‐programmes‐and‐campaigns/quick‐reads/). These are short, accessible books designed for adults with low reading ability or those who do not read often. We chose 24 unfamiliar extracts. Extracts were sampled verbatim from the book's first page, controlled for reading ease (min = 75; max = 85) and word count (range = 98 to 123). We also developed inferential multiple‐choice questions for each text presented. We piloted these questions with neurotypical adults to ensure the right level of difficulty. We presented questions without sight of the text, removing questions that could be answered through general knowledge alone, and then also assessed that when the text was provided questions could be answered correctly.

#### 2.4.4 Trait Reading Enjoyment

The current study used a single item to assess trait reading enjoyment. Participants rated a single question (‘I enjoy reading’) on a 1–9 scale. Higher numbers represent higher reading enjoyment. This measure has been found to correlate with the Adult Motivation for Reading Scale (Schutte and Malouff 2007; Bains et al. 2023a, 2023b) and is therefore considered a good and effective proxy for the trait of reading enjoyment.

### 2.5 Procedure

The task was presented using www.gorilla.sc, an online experiment platform (Anwyl‐Irvine et al. 2020). Access was restricted to those using tablets and computers. Participants gave written informed consent prior to participation. After providing demographic details, participants completed the willingness‐to‐wait task. Participants recruited via Prolific completed the reading tasks prior to participation; other participants completed these after the main experimental task. Finally, we asked participants to rate their overall reading enjoyment, followed by a short debrief.

### 2.6 Analysis

All analyses were performed using R (R Core Team 2020). Mixed‐effect logistic regression models were conducted using the lme4 package (Bates et al. 2015). Plots were generated using the effects package for R (Fox and Hong 2009). Any items that participants were familiar with were removed; 23 trials from 15 participants were removed on this basis. The data and scripts for generating models can be found at (https://doi.org/10.17605/OSF.IO/EJK9R).

#### 2.6.1 Decision to Wait

We hypothesised that a participant's decision to wait would be influenced by their reading enjoyment for that text, with higher enjoyment ratings being associated with a greater likelihood to deciding to wait to read more in adults with and without dyslexia. To address these hypotheses, we fit a mixed‐effects logistic regression model with decision to wait as the dependent variable. Fixed effects included enjoyment (mean‐centred), group and the interaction between group and enjoyment. Random intercepts of the item (extract) and participant were included to account for random variation by item and individual.

$$\text{Wait choice}\sim 1+\text{Group}\times \text{Enjoyment}+\left(1\;|\;\text{Participant}\right)+\left(1\;|\;\text{Extract}\right)$$

#### 2.6.2 Comprehension

We hypothesised that higher enjoyment would be associated with higher comprehension of the extracts in adults with and without dyslexia. To assess this, we fit a mixed‐effect logistic regression model with comprehension as the dependent variable. Enjoyment (mean‐centred), group and the interaction between them were modelled as fixed effects. Random intercepts of the item (extract) and participant were included. The second model was

$$\text{Comprehension}\sim 1+\text{Enjoyment}\times \text{Group}+\left(1\;|\;\text{Participant}\right)+\left(1\;|\;\text{Extract}\right)$$

## 3 Results

Demographic details for participants are reported in Table 1. Groups were matched on gender and age. As expected, participants with dyslexia had lower scores on the ARHQ and the sentence verification task.

**TABLE 1:** Demographic details for neurotypical adults and adults with dyslexia. Standard deviations are indicated in brackets.

	Neurotypical adults	Adults with dyslexia	
Age	24.08 (3.27)	23.44 (3.49)	*t*(115.87) = 1.04, *p* = 0.299
Gender	35F:23M:1NB	35F:24M:0NB	—
ARHQ‐Brief	0.21 (0.18)	0.63 (0.17)	*t*(115.96) = 13.18, *p* < 0.001
Sentence verification	55.08 (9.59)	45.58 (10.80)	*t*(114.39) = 5.06, *p* < 0.001

**FIGURE 1:**
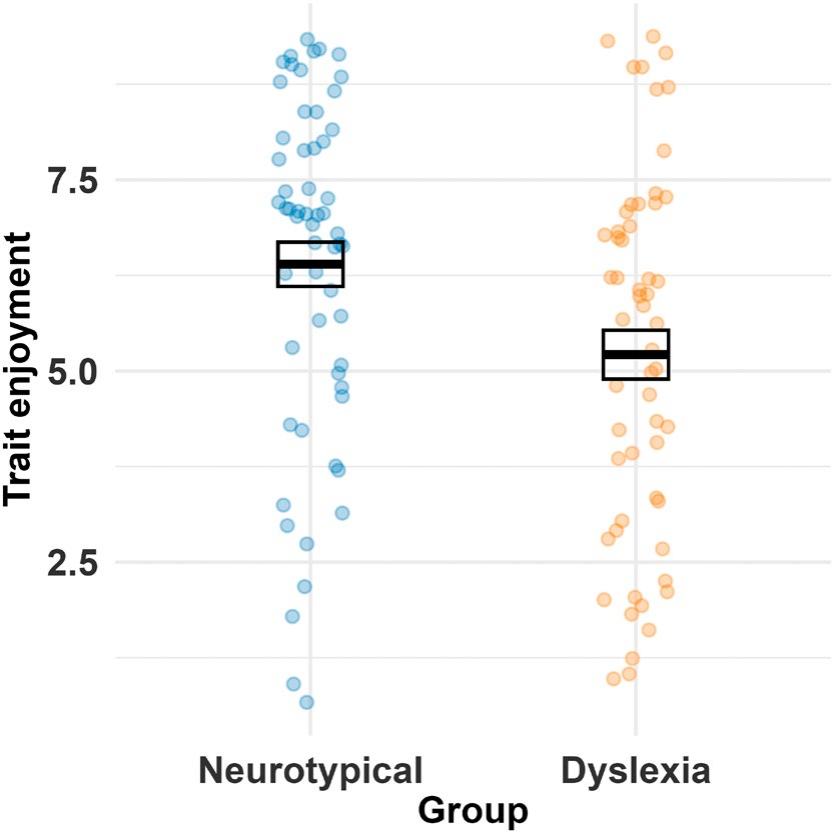
Trait reading enjoyment differs by group. The solid black line shows the group mean, and the box depicts ±1 standard error. Dots represent individual data points (blue for neurotypical adults and orange for adults with dyslexia).

When using our trait‐based measures, in line with previous literature, we found neurotypical readers (*M* = 6.4, SD = 2.21) reported higher reading enjoyment than those with dyslexia (*M* = 5.21, SD = 2.39), *t*(110.58) = 2.74, *p* = 0.007 (Figure 1).

We then analysed the measures obtained from our experimental task. In contrast to the trait‐based measure, average state‐based reading enjoyment did not differ by group, *t*(105.48) = 0.19, *p* = 0.848, with adults with dyslexia (*M* = 5.55, SD = 1.01) and neurotypical adults (*M* = 5.52, SD = 0.73) being broadly comparable. Adults with (*M* = 6.15, SD = 7.10) and without dyslexia (*M* = 6.02, SD = 6.17) were also equally willing to wait for texts, *t*(113.8) = 0.11, *p* = 0.912, indicating roughly equivalent levels of “wanting”. Again, in keeping with predicted challenges in reading, adults with dyslexia (*M* = 12.86, SD = 5.09) answered fewer comprehension questions accurately relative to neurotypical adults (*M* = 15.75, SD = 3.59), *t*(104.2) = 3.55, *p* = 0.001.

We then assessed the evidence for our key experimental hypotheses.

Hypothesis  1 *Higher enjoyment ratings will be associated with a greater likelihood of waiting to read more*.

**FIGURE 2:**
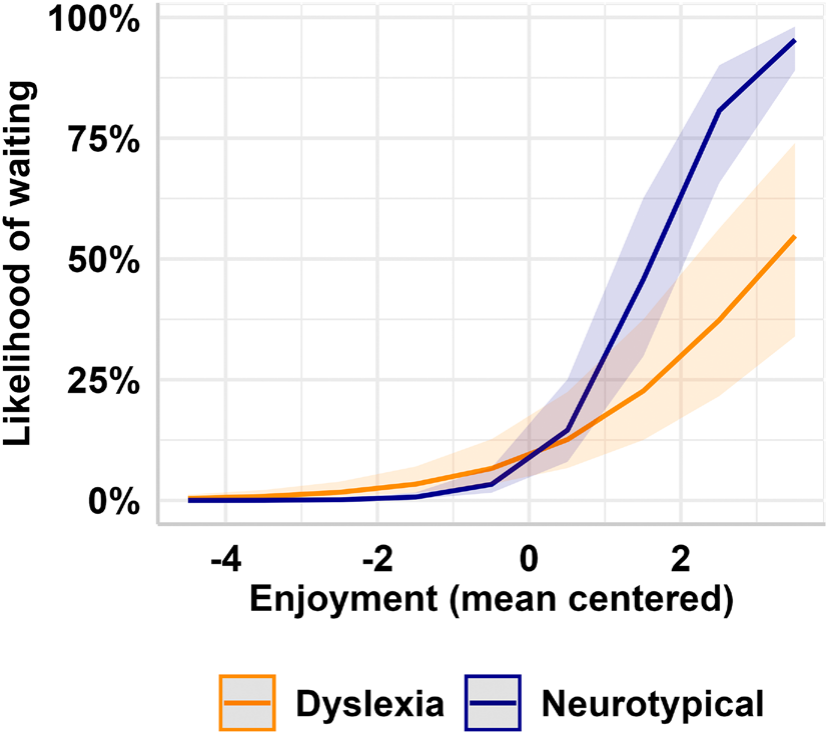
Participants were more likely to wait for the next extract when they expressed high enjoyment. Neurotypical adults were more likely to wait (illustrated in blue). This relationship was shallower than participants with dyslexia (illustrated in orange). Bands around the solid lines indicate the 95% confidence interval.

As predicted, enjoyment of an extract positively predicted participants' likelihood to wait to read more of the book, *β* = 0.71, SE = 0.085, *z* = 8.38, *p* < 0.001. In other words, if a participant enjoyed reading the presented extract, they were more likely to wait for to read more of the text. Group did not emerge as a significant main effect, *β* = −0.28, SE = 0.496, *z* = −0.568, *p* = 0.57. However, there was a significant interaction between group and enjoyment, *β* = 0.89, SE = 0.151, *z* = 5.88, *p* < 0.001. To unpack this interaction, we tested the relationship between enjoyment and waiting in each group. In adults with and without dyslexia, enjoyment was positively associated with waiting. The slope of the relationship between enjoyment and likelihood of waiting was steeper in neurotypical adults (Figure 2). In other words, a 1SD increase in enjoyment was associated with neurotypical adults being 5.32 times more likely to wait (*β* = 1.64, SE < 0.001, *z* = 1746, *p* < 0.001), while those with dyslexia were only 1.95 times more likely to wait to read more (*β* = 0.71, SE = 0.08, *z* = 8.41, *p* < 0.001). This showed that reading ability modulated the effect of enjoyment on future motivation.

Hypothesis  2 *Higher enjoyment ratings will be associated with better comprehension of extracts*.

**FIGURE 3:**
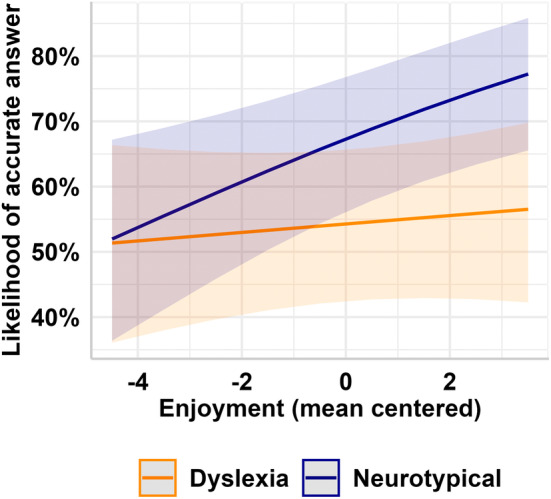
Group (neurotypical/dyslexia) modulated the relationship between enjoyment (centred) and the likelihood of answering correctly. Neurotypical adults were more likely to answer a question accurately when they enjoyed an extract (illustrated in blue). In participants with dyslexia, enjoyment did not predict accuracy (illustrated in orange). Shaded bands around the solid lines indicate the 95% confidence interval.

Somewhat to our surprise, enjoyment did not emerge as a significant predictor of comprehension, *β* = 0.03, SE = 0.05, *z* = 0.56, *p* = 0.575. However, group membership was significantly associated with accuracy (*β* = 0.55, SE = 0.17, *z* = 3.19, *p* = 0.001), and there was a trend for an interaction between group and enjoyment (*β* = 0.12, SE = 0.07, *z* = 1.76, *p* = 0.079).

When examining the effect of enjoyment in each group independently (Figure 3), enjoyment significantly predicted the likelihood of a higher comprehension accuracy within neurotypical adults, *β* = 0.14, SE = 0.05, *z* = 2.82, *p* = 0.005, replicating our previous finding. A 1SD increase in enjoyment was associated with a 1.15 times increase in the likelihood of answering the comprehension question accurately in neurotypical adults. For those with dyslexia, however, enjoyment did not predict the likelihood of comprehension accuracy, *β* = 0.039, SE = 0.05, *z* = 0.83, *p* = 0.41.

## 4 Discussion

In this study, consistent with previous work using trait‐based measures (Polychroni, Koukoura, and Anagnostou 2006; Soriano‐Ferrer and Morte‐Soriano 2017), we found that participants with dyslexia self‐reported lower reading enjoyment. Yet, our state‐based measures painted a different picture, offering insight into what might drive decisions to read. Adults with and without dyslexia showed equal levels of state‐based enjoyment when encountering a variety of texts, suggesting that they found these equally pleasurable. However, adults with dyslexia were less likely to accept the cost of waiting to continue to read.

Despite similar levels of enjoyment, we observed systematic differences in willingness to wait in the two groups. In other words, having dyslexia modulated the relationship between experiencing enjoyment for specific texts and being motivated to pursue further reading. This is likely to be because having dyslexia is likely to amplify costs associated with further reading. These costs downweigh the subjective value assigned to a stimulus (Husain and Roiser 2018). For example, the need to exert greater physical effort reduces the value assigned to a reward (Chong et al. 2017; Matthews et al. 2023). For participants with a history of reading challenges, this suggests that higher enjoyment or lower costs is necessary to produce the same motivation behaviour. Costs associated with reading could be reduced through targeted interventions to improve reading ability or reduce the difficulty of the text (Castles, Rastle, and Nation 2018; Nation, Dawson, and Hsiao 2022). Altering state‐based factors, such as the content and nature of the text, providing choice (Bains et al. 2023b), and changing the purpose of reading could help to increase enjoyment and thereby motivation. Future studies using state‐based experiments could provide helpful insight into how text and situation specific factors influence reading motivation.

Our findings also advance our previous work. In our previous study, participants were given the choice to view a book cover, as this provided participants information to seek out the book (Bains et al. 2023a). Here, we changed the decision to involve reading more text, increasing face validity. Our results were in line with previous findings—when participants enjoyed reading a text, they were more likely to wait to read more. This suggests that experiencing intrinsic reward triggers new cycles of motivation, both for information seeking and for reading more. This finding is consistent with several studies that have linked states of curiosity or intrinsic reward to greater learning and information seeking (Bains et al. 2023a, 2023b; Fandakova and Gruber 2021; Garvin and Krishnan 2022; Gruber and Ranganath 2019; Marvin and Shohamy 2016; Ripollés et al. 2018). Mechanistically, this reward signal serves to increase attention, information seeking, and, in turn, memory through the modulation of dopaminergic pathways (Gruber and Ranganath 2019; Ripollés et al. 2018). Anecdotally, we found that some participants contacted researchers to find out which books the text were drawn from, suggesting the effects of intrinsic reward outlasted the experiment.

Neurotypical participants also show an increase in comprehension when they enjoy texts, in line with our previous findings (Bains et al. 2023a, 2023b). This is unlikely to be due to a temporary boost in the ability of these skilled adult readers. Rather, we hypothesise that enjoyment may evoke greater arousal and attention, which would be associated with higher accuracy. However, in adults with dyslexia, we surprisingly did not see evidence for this boost. One possibility is that greater comprehension leads to greater enjoyment (van Bergen et al. 2023). However, we feel that it is an insufficient explanation for our data, as overall levels of enjoyment were balanced across our groups. Another option is that akin to willingness‐to‐wait, the levels of enjoyment required to trigger the value‐guided attentional mechanisms (Anderson 2019; Anderson et al. 2016) that enable better comprehension are simply higher in participants with dyslexia. Indeed, some evidence for this comes from a study investigating curiosity and learning, which demonstrated a reduced effect of information prediction error on memory in adults with dyslexia (Garvin and Krishnan 2022). Another possibility is that adults with dyslexia might experience greater uncertainty when reading, which would result in poorer meta‐cognition, which in turn might limit the benefits of reward.

Taken together, our findings suggest a need to consider the importance of reading effort or costs when considering motivation, in line with educational theories such as the expectancy‐value theory (Eccles and Wigfield 2020; Wigfield 1994). Reading environments that neurotypical adults find motivating will be perceived as more challenging for individuals with dyslexia and will elicit lower levels of enjoyment. This may not be due to the texts or reading demands themselves. Indeed, in our case, the Quick Reads series is perceived to be dyslexia friendly, and this is borne out by similar levels of enjoyment across groups. People's expectations about their reading success and perceived effort for reading might strongly modulate their future decisions. Practically, this suggests that providing more information about goals, and building programmes which allow for success, could counter these expectations about effort. Theoretically, in other domains, quantifying the trade‐offs between costs and benefits at the algorithmic level has led to insight into social, physical and cognitive decision‐making (Chong et al. 2017; Lockwood et al. 2017, 2022; Lockwood and Klein‐Flügge 2021; Matthews et al.  2023; Müller and Apps 2019). Our work, therefore, points to a strong need to be able to quantify reading effort and understand how this is evaluated in cost–benefit valuations about reading.

There are some limitations of this work. For example, we included those who self‐reported dyslexia diagnoses. Self‐selection bias may be particularly potent in the dyslexia group, creating a particular profile. For example, one participant who completed the study noted how difficult they found it, even though they were an avid reader. Thus, raising questions about the ones who did not finish the task or decide not to take part at all due to reading avoidance. Further research needs to investigate whether these behavioural patterns are consistent in those with dyslexia, either through increased compensation for task difficulty or through an intervention program.

In this study, we have demonstrated that the willingness‐to‐wait paradigm can be used to measure reward and motivation. Using the willingness‐to‐wait paradigm will allow greater experimental manipulation for reading research to explore untapped relationships. For example, it would be interesting to see if readers with dyslexia would be more willing to wait for simpler texts when informed that these will be simple.

## 5 Conclusion

In conclusion, our use of state‐based measures of enjoyment revealed a more nuanced picture of reading motivation than the sole use of trait‐based measures, distinguishing between intrinsic reward and costs. Unlike trait‐based measures, state‐based levels of enjoyment were comparable in adults with and without dyslexia, suggesting that intrinsic reward elicited by reading text was comparable across groups. Yet, the link between enjoyment and further motivation was attenuated in dyslexia, suggesting that heightened reading effort might be devaluing the rewardingness of the text. Additionally, heightened states of enjoyment were not linked to comprehension boosts in participants with dyslexia, suggesting that merely boosting enjoyment would not necessarily lead to better comprehension. Heightened states of enjoyment were associated with decisions such as continuing to read, suggesting that enjoyment might still be a useful target for building engagement.

## Ethics Statement

This study received ethics approval from the university ethics committee at Royal Holloway, University of London.

## Conflicts of Interest

The authors declare no conflicts of interest.

## Data Availability

The data that support the findings of this study are openly available in OSF at https://osf.io/ejk9r/.
